# Medical College and Hospital Notes

**Published:** 1898-01

**Authors:** 


### Medical College and Hospital Notes.

THE Woman's Hospital, which has been for more than forty years a landmark in old New York, occupying the conspicuous block at Park avenue and 49th street, has at last decided to move. The block between 49th and 50th streets and Park avenue, on which the Woman's Hospital stands, therefore, comes upon the market. The directors hope, as soon as their financial arrangements are completed, to erect more modern buildings on the new site in Eighth avenue, facing the park, and to be enabled to accommodate a great many more patients.

This institution was the pioneer in its special branch of beneficent surgery. It has done incalculable good to the city, and, with enlarged facilities, may be expected to remain in the front rank among the benevolent institutions of which New York is most proud.

THE sons of the late Henry W. Sage have presented the residence of the great philanthropist together with an endowment fund of $100,000 to Cornell University for hospital purposes. This latest gift, says the *Mail and Express*, which provides a splendid infirmary for the students of the university, is in the highest degree worthy of the long list of benefactions which have so conspicuously identified the name of Sage with the institutions of Cornell, and aside from the humane and benevolent purposes which it is destined to serve, it will be an invaluable addition to the practical organisation of the establishment as a whole. The jealous and almost fatherly interest of the elder Sage in the gradual development of the Cornell foundation has been proudly cherished by his sons, who, in this additional gift, valued in the aggregate at $200,000, have paid a beautiful tribute to the good man whose honored name they bear so worthily and so well.

---

THE new Massachusetts Hospital for consumptives and tuberculous patients at Rutland, in Worcester county, says the *Evening Post*, will be opened for patients soon after New Year's. It was built under an act of 1895. Rutland was chosen as the site because of its excellent hygienic conditions. The buildings are about 1,200 feet above the sea level, and the conditions which are most favorable for consumptive patients, are found abundantly. The hospital will have accommodations for about 150 patients, and it is probable that the rooms will be filled in a short time, for there is no limit, anywhere near that number, to those who might be suitable candidates for admission. For the most part the inmates will be people who are too poor to care for themselves, representatives of the tenement-house population.

Dr. Abbott, Secretary of the State Board of Health, says that no special method of treatment will be followed at the hospital. It is not a place for experiments, and has not been established for the benefit of any particular school of medicine, nor for the sake of establishing any particular theory. The buildings consist of an administration building in the center of a group of low, one-story wooden buildings, arranged upon the arc of a circle, and alternately long and short, in order that free access may be given to the sunlight. The buildings on one side of the group are for men and the others are for women. Each long building has seven private wards, 9x12 feet, with open fireplaces ; also a general ward, 25x90 feet, with accommodations for 22 patients, each such ward having large brick fireplaces and a ventilating shaft. There is also a "sun-room," composed wholly of glass, 12x27 feet. This is surrounded by a wide veranda. In the small buildings the general wards have only ten beds each. These wards also have their sun-rooms.

It is not expected that any regulations will be made to separate the patients from other people, although it is admitted that consumption is a contagious disease. Where there is so much open air and light, with fresh winds and purity generally, it is felt that the bacilli of the disease have little chance. Sunshine and air are in themselves destructive to disease germs. Two private hospitals for consumptives have existed in Boston for years, and last winter some neighbors made an onset upon the legislature to compel their removal, but though they had the support of two specialists, who testified for a fee of $50 each that the hospitals were a menace to the health of the neighborhood, the proof was so strong the other way that the effort was a failure. Dr. Abbott says that such hospitals are not the slightest danger to the neighborhood, as is proved by the long existence of several in the crowded parts of London.

---

During the recent slight fever scare in Memphis the students of the Memphis Hospital Medical College becoming panicky it was deemed advisable to take them away for a few days. Vanderbilt University, medical department, courteously extended the privileges of their excellent school to the faculty of the Memphis institution, who availed themselves of this by sending a number of students in charge of the dean, Dr. W. B. Rogers, to Nashville for a week.—*Memphis Medical Monthly.*

---

The staff of the Erie County Hospital held its annual meeting December 20, 1897, when the following officers were elected to serve for the ensuing year: President, Dr. William C. Phelps; vice-president, Dr. Henry C. Buswell; secretary and treasurer, Dr. Earl P. Lothrop; executive committee, Drs. E. P. Lothrop, H. C. Buswell, A. A. Hubbell, E. J. Meyer, F. S. Crego and H. U. Williams.

